# MEMS Differential Pressure Sensor with Dynamic Pressure Canceler for Precision Altitude Estimation

**DOI:** 10.3390/mi14101941

**Published:** 2023-10-18

**Authors:** Shun Yasunaga, Hidetoshi Takahashi, Tomoyuki Takahata, Isao Shimoyama

**Affiliations:** 1 Department of Electrical Engineering and Information Systems, Graduate School of Engineering, The University of Tokyo, 7-3-1 Hongo, Bunkyo-ku, Tokyo 113-8656, Japan; yasunaga@if.t.u-tokyo.ac.jp; 2 Department of Mechanical Engineering, Faculty of Science and Technology, Keio University, 3-14-1 Hiyoshi, Kouhoku-ku, Yokohama 223-8522, Kanagawa, Japan; htakahashi@mech.keio.ac.jp; 3 Department of Advanced Machinery Engineering, School of Engineering, Tokyo Denki University, 5 Senju Asahi-cho, Adachi-ku, Tokyo 120-8551, Japan; t-tkht@mail.dendai.ac.jp; 4 Imizu Campus, Toyama Prefectural University, 5180 Kurokawa, Imizu 939-0398, Toyama, Japan; 5 Hongo Campus, The University of Tokyo, 7-3-1 Hongo, Bunkyo-ku, Tokyo 113-8654, Japan

**Keywords:** MEMS, pressure sensor, wind effect, altitude estimation

## Abstract

Atmospheric pressure measurements based on microelectromechanical systems (MEMSs) can extend accessibility to altitude information. A differential pressure sensor using a thin cantilever and an air chamber is a promising sensing element for sub-centimeter resolution. However, its vulnerability to wind and the lack of height estimation algorithms for real-time operation are issues that remain to be solved. We propose a sensor “cap” that cancels the wind effect and noise by utilizing the airflow around a sphere. A set of holes on the spherical cap transmits only the atmospheric pressure to the sensor. In addition, we have developed a height estimation method based on a discrete transfer function model. As a result, both dynamic pressure and noise are suppressed, and height is estimated under a 5 m/s wind, reconstructing the trajectory with an estimation error of 2.8 cm. The developed sensing system enhances height information in outdoor applications such as unmanned aerial vehicles and wave height measurements.

## 1. Introduction

Altitude is one of the most important indices for localizing mobile agents and space mapping [1]. Conventional methods for measuring altitude include integrating accelerometer responses [2,3], calculations from radar measurements [4,5], and measuring the time of flight of light or sound reflecting off an object or the ground [6]. We can also utilize camera parallax [7,8] and global positioning system (GPS) information [9,10,11]. Estimating altitude from atmospheric pressure [2,3,12,13,14,15] is another method that can be used to acquire altitude information. Each method has its proper place and time of use, and among them, atmospheric pressure is a measurable physical quantity suitable for low-power altitude measurements in environments without reference for altitude, such as in the air or for ocean waves. Attitude estimation of unmanned aerial vehicles (UAVs) [6,13] and wave height measurements at sea [9,16], for example, can benefit from atmospheric-pressure-based measurements.

Previously, we developed a cantilever-type barometric sensor based on microelectromechanical systems (MEMSs) [17,18,19]. The sensor consists of a MEMS cantilever as the sensing element and an air chamber attached under the sensor chip. Altitude estimation based on this sensor can provide a resolution of less than 1 cm in height and has a fast response in the order of milliseconds [20], owing to the small mass of the sensing element. However, two factors remain to be solved before its practical application: vulnerability to wind and an estimation method for real-time operation.

Wind is a significant factor, particularly in outdoor applications, owing to the dynamic pressure created on the surface of the sensor. Although the source of altitude information is static pressure, it is impossible to distinguish it from dynamic pressure, which is inseparably superimposed in the measurement. According to Bernoulli’s law, a wind speed of 1 m/s under standard conditions (0 °C, 1 atm) will produce a dynamic pressure of up to 0.6 Pa, which amounts to 5 cm of elevation near sea level and exceeds the altitude resolution of the sensor itself. Therefore, developing a system that can measure atmospheric pressure while suppressing the wind effect is crucial to accurately measuring air pressure [21,22].

Regarding the height estimation method, the working principle of the cantilever-type differential pressure sensor has been investigated to determine its transfer function [18,19]; however, an estimation method that allows real-time data processing involving height estimation has yet to be developed.

**Figure 1:**
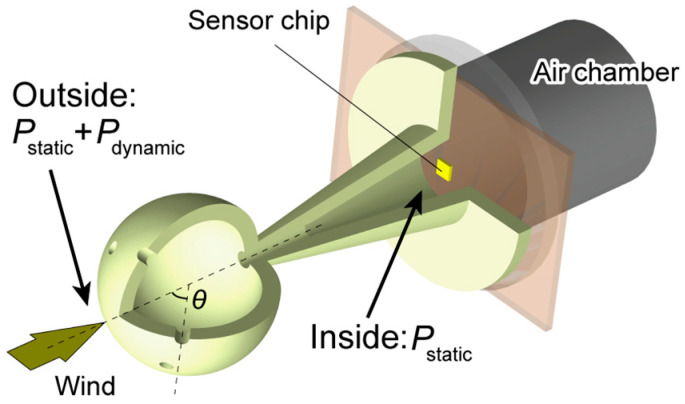
Concept of using a sensor cap to cancel dynamic pressure. At the bottom of the cap is a MEMS cantilever-type DPS attached to a printed circuit board and connected to an air chamber.

This study proposes a wind-tolerant sensor cap that can be attached to a barometric sensor for high-precision altitude measurements (Figure 1). Here, we especially focus on one-directional wind, which can be encountered, for example, on a cruising UAV that has limited load size and faces wind from the direction of travel. The sensor cap was designed and developed based on the airflow and pressure distribution on a spherical surface. By changing the positions of the holes on the sphere, we experimentally observed that dynamic pressure and noise corresponded to theoretical pressure distribution, which allowed the determination of an optimized sensor cap that suppressed the dynamic pressure and noise caused by wind. We also developed a height estimation method based on a digitalized transfer function model. Using this method, we demonstrated that height estimation is possible in the presence of wind.

## 2. Materials and Methods

### 2.1. Theory and Requirements

We can estimate the altitude from a barometric pressure sensor signal because there is a one-to-one relationship between the height above sea level, *h*, and the atmospheric pressure, *P*. The height above sea level is calculated from the atmospheric pressure using [23]

(1) $$P=P_{0}{\left(\frac{T_{0}}{T_{0}+ch}\right)}^{\frac{M_{0}g}{Rc}}.$$

*P*_0_ and *T*_0_ are the atmospheric pressure and temperature at *h* = 0, respectively; *c* is the gradient of temperature per height (−6.5 × 10^−3^ K/m); *M*_0_ is the average molar mass of air (28.8 g/mol); *g* is the acceleration due to the Earth’s gravity (9.80 m/s^2^); and *R* is the gas constant (8.31 × 10^3^ m^2^ g s^−2^ K^−1^ mol^−1^). We can use the first-order Taylor expansion around *h* = 0,

(2) $$\Delta P=-\frac{P_{0}M_{0}g}{RT_{0}}\Delta h,$$

for a displacement, Δ*h*, near sea level, with less than a 1% error for 0–200 m. Although we cannot obtain absolute pressure from this simple relationship, it provides enough information in many situations, including the aforementioned possible applications.

However, the dynamic pressure caused by airflow around a pressure sensor influences the sensor’s measurement. Dynamic pressure is a function of airflow velocity and is indistinguishably added to static (atmospheric) pressure. Moreover, the airflow around the sensor will cause local turbulence, which results in fast fluctuations (noise) in the sensor signal. Therefore, dynamic pressure and noise should be suppressed or compensated in outdoor applications or for movable platforms.

### 2.2. MEMS Pressure Sensor Element

General barometric sensors, called absolute pressure sensors (APSs), directly read atmospheric pressure values. Their operating principle is that the difference between the pressure of a reference air chamber and the external pressure will deform a diaphragm, and the extent of the deformation will indicate the atmospheric pressure [14]. Although an APS has a wide measurement range, it requires a robust structure that can withstand the maximum expected pressure, thus sacrificing sensitivity.

**Figure 2:**
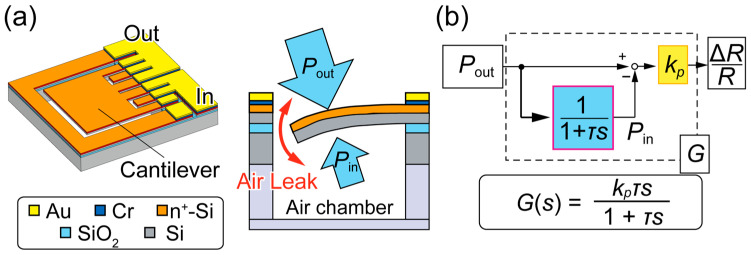
Concept of MEMS cantilever pressure sensor: (**a**) schematic and (**b**) transfer function model.

In this study, we used an MEMS differential pressure sensor (DPS) as a sensing element that was made sensitive without the need for robustness (Figure 2a). A thin cantilever, made using a silicon-on-insulator (SOI) wafer with a sub-μm-thick silicon device layer, sensed the differential pressure across a sensor chip. The differential pressure between the top and lower sides deformed the cantilever, which was detected as a change in the resistance of the piezoresistor, formed on the top side of the cantilever by doping. The cantilever legs were narrowed to concentrate the strain, resulting in a large resistance change.

When an air chamber is attached to one side of the DPS, the cantilever will deform according to the differential pressure between the external (outside) pressure, *P*_out_, and the internal chamber pressure, *P*_in_. When *P*_out_ is atmospheric, the DPS will function as a pressure “change” sensor that can detect an ambient pressure change within a certain time. Hereafter, we define differential pressure as Δ*P* = *P*_out_ − *P*_in_. Unlike the diaphragm-type APS, the cantilever-type DPS allows airflow through the gap around the cantilever until the differential pressure vanishes (*P*_in_ reaches *P*_out_). The combination of the DPS and the chamber has been identified as a first-order high-pass filter [19], with the transfer function from *P*_out_ to Δ*P* being *τs*/(1 + *τs*), where *τ* is the time constant of the system (Figure 2b). The time constant is a measure of time it takes for *P*_in_ to reach *P*_out_. The differential pressure is transduced to the fractional resistance change, Δ*R*/*R*, at the sensor sensitivity rate, *k*_p_, followed by conversion to a voltage and amplification with an amplifier gain, *k*_amp_, before readout. Therefore, the transfer function of the sensing system, *G*(*s*), is expressed as

(3) $$G\left(s\right)=\frac{k_{\mathrm{amp}}k_{p}\tau s}{1+\tau s}.$$

The working principle of the DPS, in which the pressure inside the air chamber follows that of the outside by allowing air leakage through the gap, indicates that the time constant is a function of the chamber volume (a larger chamber requires a longer time constant and vice versa). This air leak limits the maximum differential pressure exerted on the cantilever surface. Unlike an APS, which would use a sealed chamber as a pressure reference and thus require a sensing structure sufficiently robust enough to withstand the unlimited differential pressure at the cost of sensitivity, the pressure reference of the DPS always stays close to the outside pressure, owing to the air leak at the gap, which allows for a thin, sensitive structure. We can also benefit from the thin structure in terms of its resonance frequency, which is high enough in the order of 10 kHz [20] to ensure accurate measurement of much slower pressure changes in height estimations.

### 2.3. Sensor Cap

To eliminate the dynamic pressure and noise caused by wind, we devised a sensor “cap”. The dynamic pressure could be suppressed if we conducted a measurement at the exact point where the pressure is always equal to the static pressure regardless of the wind speed. A sphere is a good candidate for this purpose because of its omnidirectional shape and the predictable pressure distribution on its surface.

Considering the flow around a sphere, the pressure is the highest at the meeting point of the streamline, where the flow is stagnant, gradually decreasing to below atmospheric pressure as air flows faster around the surface. The theoretical pressure distribution, *P*, for laminar flow as a function of the angle from the streamline (*θ*) is given as [24]

(4) $$P-P_{0}=\frac{1}{2}\rho v^{2}\left(1-\frac{9}{4}\sin^{2}\theta \right),$$

where *P*_0_ is the atmospheric (static) pressure, *ρ* is the air density, and *v* is the flow velocity. Although experiments have shown slightly different distributions than this theoretical calculation [25], in either case, the pressure around the sphere will reach the atmospheric pressure at a certain angle, *θ*_0_, which is less than 90° (41.9° for laminar flow Equation (4)), regardless of the flow velocity. If we can extract and measure the pressure at this particular point, it will always be equal to the static pressure, even in the wind.

The proposed sensor cap has a spherical head with multiple evenly placed holes, each located at the same angle, *θ*, from the streamline, introducing pressure into the inside of the sensor cap. The effect of using multiple holes is that the cap averages the pressure from each hole to produce a less noisy pressure that is fed to the sensor. The spherical head is supported by a hollow pillar that separates the cap’s head (where the pressure to be measured is taken in) from the rest of the sensor assembly, which would disturb the airflow if placed nearby. The pillar allows the pressure from the sphere to be transmitted to the MEMS cantilever pressure sensor.

### 2.4. Discrete Transfer Function Model for Height Estimation

We could obtain height information from the sensor output by applying the inverse transfer function *G*^−1^(*s*) = (1 + *τs*)/*k*_amp_*k*_p_*τs*. We employed the bilinear *z*-transform to convert the transfer function of the continuous system into its discrete counterpart,

(5) $$s=\frac{2}{T}\frac{1-z^{-1}}{1+z^{-1}},$$

where *T* is the sampling period. The time constant for the discrete system is then

(6) $$\frac{1}{\tau }=\frac{2}{T}\tan\left(\frac{T}{2\tau^{\prime }}\right),$$

where *τ*^′^ is the corrected time constant for the discrete system. Therefore, the transfer function for the discrete system is

(7) $$G^{-1}\left(z\right)=\frac{\left(T+2\tau^{\prime }\right)+\left(T-2\tau^{\prime }\right)z^{-1}}{2k_{\mathrm{amp}}k_{p}\tau^{\prime }\left(1-z^{-1}\right)}.$$

The outside pressure change, *P*_out_, at data point *n* (the sampling interval is *T*) can be calculated from the measured data, *S*(*n*), using

(8) $$P_{\mathrm{out}}\left(n\right)=P_{\mathrm{out}}\left(n-1\right)+\frac{1}{k_{\mathrm{amp}}k_{p}}\left\{\left(S\left(n\right)-S\left(n-1\right)\right)+\frac{T}{\tau^{\prime }}\frac{S\left(n\right)+S\left(n-1\right)}{2}\right\}.$$

This formula updates the pressure estimation.

## 3. Results

### 3.1. Fabrication of Sensing Elements

**Figure 3:**
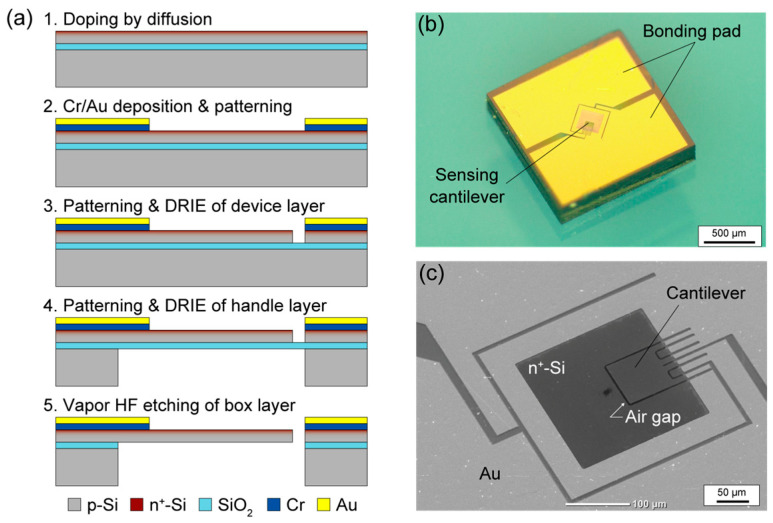
MEMS cantilever differential pressure sensor: (**a**) fabrication process, (**b**) overall appearance, and (**c**) SEM photograph.

The fabrication process of the MEMS cantilever-type DPS followed that of previous research using an SOI wafer [20] (Figure 3a). First, a 300 nm-thick silicon device layer was doped using rapid thermal diffusion to form a piezoresistive layer. Chromium and gold layers were then deposited using evaporation and were patterned using photolithography and wet etching. The device layer was then etched to form a cantilever shape using deep reactive ion etching (DRIE). Finally, DRIE was used to etch the handle silicon layer from the back side, and the oxide layer was removed using hydrogen fluoride vapor. Figure 3b,c show the fabricated sensor chip. The cantilever was 100 µm in width and length and surrounded by a 3 µm-wide gap. Before the loading of the sensor chip, a through-hole (1 mm in diameter) was drilled through a printed circuit board (PCB). The fabricated sensor chip was then glued onto the PCB at the through-hole using epoxy resin and wire bonding.

### 3.2. Sensor System Development

**Figure 4:**
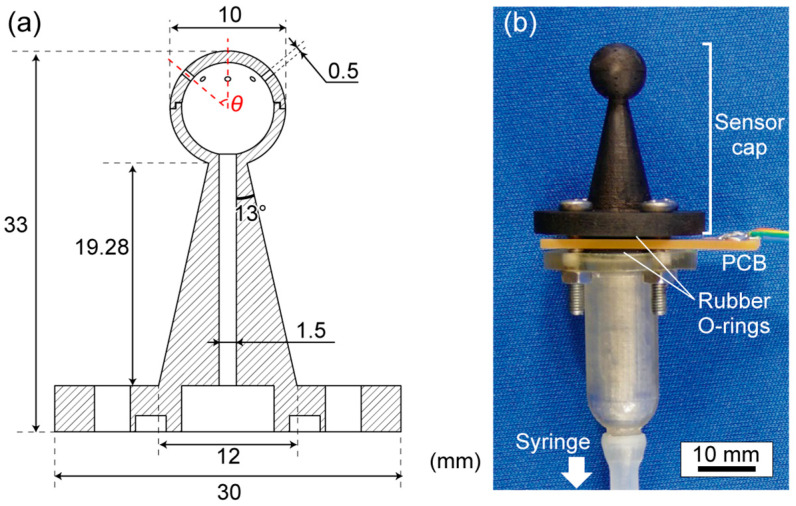
(**a**) Cap design and (**b**) fabricated sensor cap joined with PCB using O-rings and showing the tube and connector to a syringe to allow variable-volume tests.

Figure 4 illustrates the sensor cap we designed in this study. Sensing platforms for mounting pressure sensors, such as unmanned aerial vehicles and buoys, are limited in size and payload. In designing the sensor cap, the requirements are that (1) the size should be small enough not to interfere with the performance of the platform itself and (2) the region in which to measure the pressure should be small so that any gravity-induced pressure change is of the same order as the sensor’s pressure resolution. Based on these requirements, we set the sphere size to be 10 mm in diameter, comparable to the height resolution of the DPS. A 20 mm-long hollow pillar that transmitted the pressure to the sensor chip separated the sphere from the rest of the sensor system, which disturbed the airflow. On the sphere, eight holes, each with a 0.5 mm diameter, were placed at a specific angle, *θ*, from the streamline to take in the pressure. The holes were sufficiently smaller than the surface area of the sphere so as not to disturb the flow but large enough for the pressure transmission. The number of holes was set to eight to obtain the effect of averaging signals for noise reduction while ensuring most of the flow would pass over the smooth surface of the sphere. We varied *θ* from 0° to 75° (note that the cap for when *θ* = 0° had only one hole) to experimentally obtain the optimal angle. These sensor caps were fabricated using a photopolymerizing 3D printer (EDEN260 V, Stratasys Ltd., Eden Prairie, Minnesota, USA; resin: Full-cure720), and the pillar of the sensor cap was painted black to eliminate the influence of light on the photosensitive piezoresistors.

If the holes on the sphere were too small or the volume inside the cap was too large, the transmission of pressure to the sensor chip would be delayed. The effect of the sensor cap on the delay of the pressure transmission was estimated as follows: The total inlet area of the eight circular holes was 150 times the area of the cantilever’s surface and the gap combined (amounting to the maximum through-hole area on the sensor chip when the cantilever was fully bent). The sensor cap itself had an internal volume of 0.5 mL. Assuming that the flow was proportional to the cross-sectional area, the pressure inside the sensor cap reached equilibrium 150 × (*V* [mL]/0.5 [mL]) times faster than it would take for a *V* mL-chamber to reach the outside pressure by air leakage through the cantilever sensor chip. Therefore, the signal delay at the sensor cap was negligible, and we could assume that the pressure in the sensor cap was equal to the atmospheric pressure if the air chamber volume were comparable to or larger than 0.5 mL.

We prepared two types of air chambers: a rigid chamber with a fixed volume and a variable-volume chamber implemented using a syringe with a tube and a connector. The fixed-volume chamber and the connector with the variable volume were 3D-printed. The sensor cap, the PCB to hold the sensor chip in place, and the air chamber were connected by rubber O-rings and fastened using four screws. Figure 4b shows the completed sensor.

### 3.3. Sensor System Characterization

We first characterized the sensor by measuring the static and dynamic responses. According to Equation (3), the proposed sensor system is modeled based on two parameters: sensor sensitivity, *k*_p_ (=(Δ*R*/*R*)/Δ*P*), and the time constant, *τ* [19]. We evaluated the sensor sensitivity by applying a constant differential pressure across the sensor chip and the time constant by using a step pressure input. The amplification gain, *k*_amp_, in Equation (3) was 250 throughout this study, where the sensor output, as Δ*R*/*R*, was measured using a one-gauge Wheatstone bridge circuit (bridge voltage = 1 V) and an instrumentation amplifier with a gain of 1000.

**Figure 5:**
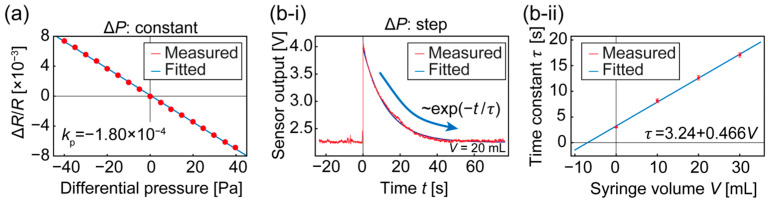
System calibration curves. (**a**) Sensitivity of sensor chip and (**b**-**i**,**b**-**ii**) dynamic characteristics of the system (time-series response (**b**-**i**) and obtained time constant vs. chamber volume (**b**-**ii**)). Error bars in (**b**-**ii**) correspond to the mean value ± the standard deviation for ten measurements.

Figure 5a shows the measured Δ*R*/*R* versus the applied differential pressure, up to ±40 Pa, using a pressure calibrator (KAL100; Halstrup-Walcher GmbH, Kirchzarten, Germany). The gradient in the plot corresponds to the sensor sensitivity, *k*_p_. The result shows a linear relationship in the measured range, where *k*_p_ = −1.80 × 10^−4^ Pa^−1^.

The time constant, *τ*, of the system was measured using a step input and several chamber volumes (using the syringe arrangement). The step input was emulated in the measurement by manually opening a valve that had confined the sensor system under compressed air. Theoretically, the sensor system modeled in Equation (3) responds to a step input of *P*_out_(*t*) = *P*_step_*u*(*t*) exponentially as *k*_p_*P*_step_exp(−*t*/*τ*), where

(9) $$u\left(t\right)=\left\{\begin{array}{c} 0\;(t<0)\\ 1\;\left(t\geq 0\right) \end{array}\right..$$

The measured responses shown in Figure 5b-i agree with this theory. We obtained the time constant by fitting an exponential curve to the response 0.2 s after opening the valve to remove the effect of unideal step input. The results for several chamber volumes, shown in Figure 5b-ii, were in a proportional relationship that was compatible with the working principle that air leakage allows inside pressure to eventually become equal to outside pressure [19]. Note that the horizontal axis represents the volume of the syringe, excluding that of the tube and connector. The intercept of the horizontal axis should correspond to the summed volume of the tube and the connector. These results indicate that the sensor system functioned as a differential pressure sensor, obeying transfer function model Equation (3).

### 3.4. Evaluation of the Cap

**Figure 6:**
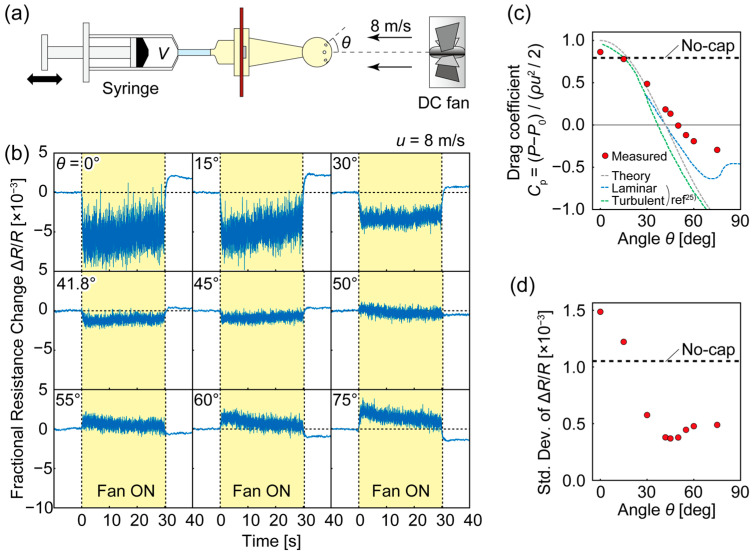
Evaluation of sensor cap: (**a**) experimental setup, (**b**) sensor output as the fan was turned on and off, (**c**) effect of dynamic pressure, and (**d**) noise vs. angle of incidence. In (**c**), the gray dashed line corresponds to the theoretical distribution on a sphere (Equation (4)), and the blue and green dashed lines correspond to experimental values obtained for laminar and turbulent flows, respectively [25].

Using the characterized DPS and the experimental setup illustrated in Figure 6a, we evaluated the performance of the sensor cap. The sensor cap was mounted on the PCB sensor assembly, and the wind was applied using a direct-current (DC) fan placed upstream of the sensor cap. The chamber was the tube-connected syringe (*V* = 100 mL), whose time constant was *τ* = 50 s. Figure 6b shows the measured responses when a wind velocity of *v* = 8 m/s was applied, kept on, and stopped for various *θ* values. When *θ* = 0°, with the pressure-taking-in hole only at the stagnant point, the sensor output dropped as the DC fan was turned on (*t* = 0 s), corresponding to an increase in *P*_out_ (because of the negative *k*_p_). The signal then increased, with noise toward the origin, as *P*_in_ followed *P*_out_ while the wind was present. After 30 s, when the DC fan was turned off and *P*_out_ became the atmospheric pressure, *P*_in_ was higher than *P*_out_, resulting in a positive sensor output higher than at the initial state.

The overall response to a change in wind speed decreased as *θ* increased to 50° and intensified again in the opposite direction for an even larger *θ*. To evaluate the effect of dynamic pressure, we fitted a line to the response *t* = 2–12 s and calculated its extrapolation to *t* = 0 s as the initial differential pressure, Δ*P*. Figure 6c shows the drag coefficient obtained from Δ*P* divided by the kinetic energy, *ρv*^2^/2, where *ρ* is the air density. The theoretical pressure distribution (Equation (4)), as well as the reported experimental values [25], are also plotted. As a result, the measured dynamic pressure as a function of *θ* followed a sinusoidal curve, corresponding to the theoretical prediction. The sensor cap with *θ* = 50° reduced the dynamic pressure the most successfully; for it, the wind effect was 0.90% of the result of the no-cap condition.

We also evaluated the noise in the airflow using this measurement. The dynamic pressure effect was compensated by subtracting the fitting line obtained from the *t* = 2–12 s response; then, the standard deviation was calculated as the noise. The result in Figure 6d is distributed similarly to the dynamic pressure amplitude in Figure 6c, implying the relationship between noise and dynamic pressure. The noise was lowest at *θ* = 45°, with 35% of that measured in the no-cap condition, and the *θ* = 50° cap, which reduced the dynamic pressure the most, suppressed the noise to 36%. From the viewpoints of both dynamic pressure reduction and noise suppression, we concluded that *θ* = 50° was the best placement of the hole to take in pressure.

### 3.5. Height Estimation Method and Demonstration

**Figure 7:**
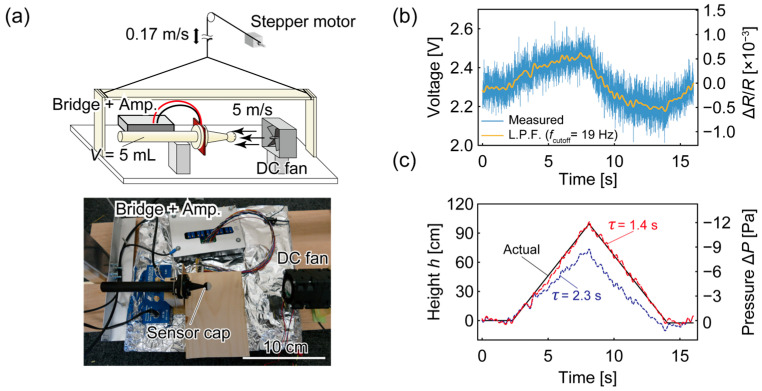
Height estimation under 5 m/s wind conditions: (**a**) experimental setup, (**b**) measured and low-pass filtered (L.P.F.) sensor output, and (**c**) height estimations vs actual height profile.

We evaluated the height estimation performance of the sensor system using the *θ* = 50° cap and a 5 m/s wind. The measurement setup with the DC fan was loaded on a platform, which was winched up and down approximately 1 m at a rate of 0.17 m/s (Figure 7a). The same sensor chip and readout circuits used in the measurements thus far were employed, and the chamber volume was set to 5 mL. The atmospheric conditions were *P*_0_ = 101 kPa and *T*_0_ = 14.8 °C, which reduced Equation (2) to

(10) $$\Delta P\;\left[\mathrm{Pa}\right]=-12\;\left[\mathrm{Pa}/m\right]\times \left(\Delta h\;\left[m\right]\right).$$

Figure 7b shows the sensor output measured at 500 Hz and the processed signal using a low-pass filter with a cutoff frequency of 19 Hz (see Discussion for the required sampling frequencies based on applications). The low-pass-filtered data were then processed using Equation (8) to obtain the pressure change, as shown in Figure 7c. The solid black line indicates the actual displacement of the platform. The blue dashed line is the estimated height using the time constant *τ* = 2.3 s, calculated from Figure 5b-ii and the chamber volume of 5 mL, and the red dashed line shows the results using *τ* = 1.4 s. The estimated profiles for the two time constants were triangular, similarly to the actual profile obtained as the platform moved. However, the estimated displacement with *τ* = 2.3 s was smaller overall. When we recalculated the trajectory with different time constant values, *τ* = 1.4 s reproduced the actual trajectory most accurately; for this, the root mean square of the estimation error was 2.8 cm.

It is implausible to attribute the difference in the time constant to sensor fabrication error because *τ* = 1.4 s corresponds to a chamber volume of 3.0 mL, which is only 61% of the actual volume used, according to the proportional relationship between the chamber volume and the time constant obtained in Figure 5b. We can reasonably assume that the time constant in this experiment was shorter than the value obtained from Figure 5b, owing to air leakage between the PCB and the air chamber. In this experiment, the air chamber was a fully 3D-printed rigid chamber instead of the syringe attached via a 3D-printed connector. Although both the rigid chamber and the connector were 3D-printed, the surface of the connector was smoother because of wearing after many attach/detach operations and the orientation during printing. In general, photopolymerizing 3D printers produce rougher surfaces on the vertical face than on the horizontal, and the connector was printed horizontally whereas the rigid chamber was printed vertically. The rougher surface of the rigid chamber, which was not completely sealed by the rubber O-ring, leaked air through an alternate route than the supposedly only path for air to move in and out of the chamber, accelerating the equalization of the pressure inside and outside of the chamber. However, when air leaks through the rougher surface of the rigid chamber caused air leakage between the air chamber and the PCB in addition to the usual path across the sensor chip, the flow rate to equalize the differential pressure increased, therefore shortening the time constant.

To compare the noise unrelated to elevation for different wind speeds, the standard deviation of the measurement under 8 m/s without vertical movement was 2.84 cm after a 19 Hz low-pass filter, while for 5 m/s, it was 2.71 cm. This indicates that the effect of the wind-related fast-fluctuating noise was not dominant in the estimation, proving the effectiveness of the sensor cap in reducing the noise.

## 4. Discussion

In the sensor cap evaluation, we obtained *θ* = 50° as the best angle from streamline to place the hole to take in the pressure. This value is larger than the predicted theoretical and previously reported values [25], which might be attributable to the existence of the sensor structure after the flow moves around the sphere. Unlike an isolated sphere, the sensor assembly includes the cap’s support (for screw fastening), the PCB, and the air chamber, which cause dynamic pressure. Although the pillar separating the spherical cap head from the rest of the assembly will ease their effect, the pressure distribution around the sphere will likely be higher than that of an isolated sphere, resulting in a larger *θ*_0_.

A height estimation accuracy of 2.8 cm is comparable to the height resolutions of radiofrequency-based ultrawideband sensors [26,27], ultrasonic sensors [6], and GPS velocity estimation [11]. We have summarized the comparison to other sensing techniques in Table 1. Regarding the signal delay discussed in Section 3.2, the time constant for a 5 mL chamber, obtained in Section 3.5, was on the level of 1 s. Therefore, the delay due to the proposed sensor cap should have been on the level of 1 ms or less. This value is sufficiently low, considering the frequency characteristics of the cantilever differential-pressure sensor, which ensures accurate measurements at up to 200 Hz [20]. Therefore, the most significant factor limiting the sampling bandwidth is the low-pass filter used to eliminate measurement noise. The bandwidth should be chosen according to the specific requirements of the application and the expected noise level. For example, some signals for UAVs are sampled at 10–20 Hz [28,29], and the frequency band of maritime waves is 0.03–0.5 Hz [10,11].

As indicated by a previous study involving sensor fusion [18], MEMS cantilever pressure sensors should be used together with absolute pressure sensors to achieve the long-term stability of the sensing system. Although cantilever pressure sensors are capable of high sensitivity, they suffer from estimation drift because they are essentially a high-pass filter, and integral calculations are necessary in the estimation process. In contrast, absolute pressure sensors, including the diaphragm type, have low sensitivity but remain stable over time. By combining the proposed sensor system with the low-pass-filtered signal of an absolute pressure sensor, both long-term stability and high sensitivity can be achieved. This also provides the information of absolute altitude that might be required in some applications. In this research, we focused on the performance of the system using MEMS cantilever pressure sensors and performed a demonstration over a short period in which long-term stability was not required; however, the sensor cap can be applied to absolute pressure sensors as well. Thus, long-term precision measurements of pressure in different environments should be possible using the sensor cap.

The time constant of the sensor should be chosen according to the expected rate of pressure change and the measurable range of the cantilever in use. A long time constant, which can be realized by a large chamber or by a narrow gap around the cantilever, will have less integration-derived drift in its estimation because the pressure in the chamber will be maintained for a long period of time. However, it can cope with a smaller range of pressure change because the differential pressure must be within the linearly measurable range of the DPS. Likewise, a short time constant can be used even in a fast movement but can have more impact on the drift in estimation. A rough criterion for the choice of time constant is the linearly measurable range divided by the expected rate-of-pressure change (obtained from the maximum vertical speed and Equation (2)).

In outdoor applications, it is unrealistic to assume that wind blows in a fixed direction. To cope with this omnidirectionality, several of the proposed sensor systems can be placed in multiple directions so that the appropriate sensor can be chosen for estimation. Because the proposed sensor cap is on the level of centimeters, loading multiple sensor systems on small platforms should be possible. However, it is impossible to cover all spherical angles using a limited number of sensor systems. In this regard, we can expect that because the holes on the sphere have rotational symmetry, with evenly placed holes, the measurement will become robust against tilted streamlines, although we have not yet verified this. If a hole faces the airflow at a smaller angle, *θ*_0_, and experiences a positive dynamic pressure, the effective angle of the hole on the opposite side will be greater than *θ*_0_ and, therefore, will experience a countering negative dynamic pressure because the pressure distribution near *θ* = *θ*_0_ will nearly be linear. After the pressures are combined within the sensor cap, the dynamic pressure should still be canceled. However, when the angle grows too much, the pressure distribution will no longer be linear. In the case of imperfectly turbulent flow, for example, the flow separates from the surface at approximately *θ* = 80° [25], giving a sudden change in pressure. This situation corresponds to a tilt angle of 30° from the streamline for the 50° cap, which might be the maximum acceptable angle.

Another factor to be considered in the outdoor application is measurement robustness against changing conditions. The cantilever DPS made of crystalline silicon and gold has robust durability but can be affected by temperature because of the temperature-dependent resistivity of doped silicon [20,34]. To avoid this dependency, a temperature-compensating resistor can be introduced on the same sensor chip and incorporated in the Wheatstone bridge [35].

## 5. Conclusions

To eliminate the wind effect in pressure measurements using a cantilever-type MEMS sensor, we developed a 10 mm spherical head cap with eight holes and a supporting hollow pillar. When each of the holes on the sphere faced the streamline at 50°, the dynamic pressure and noise were suppressed to 0.9% and 36% of those measured for the no-cap condition, respectively, considering an 8 m/s wind. We also developed a height estimation formula and demonstrated its accuracy based on a reconstructed height profile of 1 m of vertical movement under 5 m/s wind conditions, yielding an estimation error of 2.8 cm. We expect that the proposed sensing system will contribute to expanding the availability of pressure-based height information in outdoor applications that require high sensitivity.

## Figures and Tables

**Table 1:** Performance compared among height-measuring techniques.

Method	Range	Differential Accuracy
Ultrasound ToF [30]	30 m	2 cm
RF ToF [27]	40 m	2.1 cm
Radar [31,32]	≈200 m	<5 cm
Absolute Pressure Sensor [33]	−730 m–9500 m ^1^	23 cm ^2^
This Work	200 m ^3^	2.8 cm

^1^ Values of 30–110 kPa, converted with Equation (1). ^2^ A value of 3.9 Pa, converted with Equation (10). ^3^ A 1% error range when using Equation (2); structurally unlimited.
